# Supplementary figures and images for: Individualistic reward-seeking strategies that predict response to nicotine emerge among isogenic male mice living in a micro-society
Source: PLoS Biol. 2024 Oct 24;22(10):e3002850. doi: 10.1371/journal.pbio.3002850 (PMC11501037; doi:10.1371/journal.pbio.3002850)

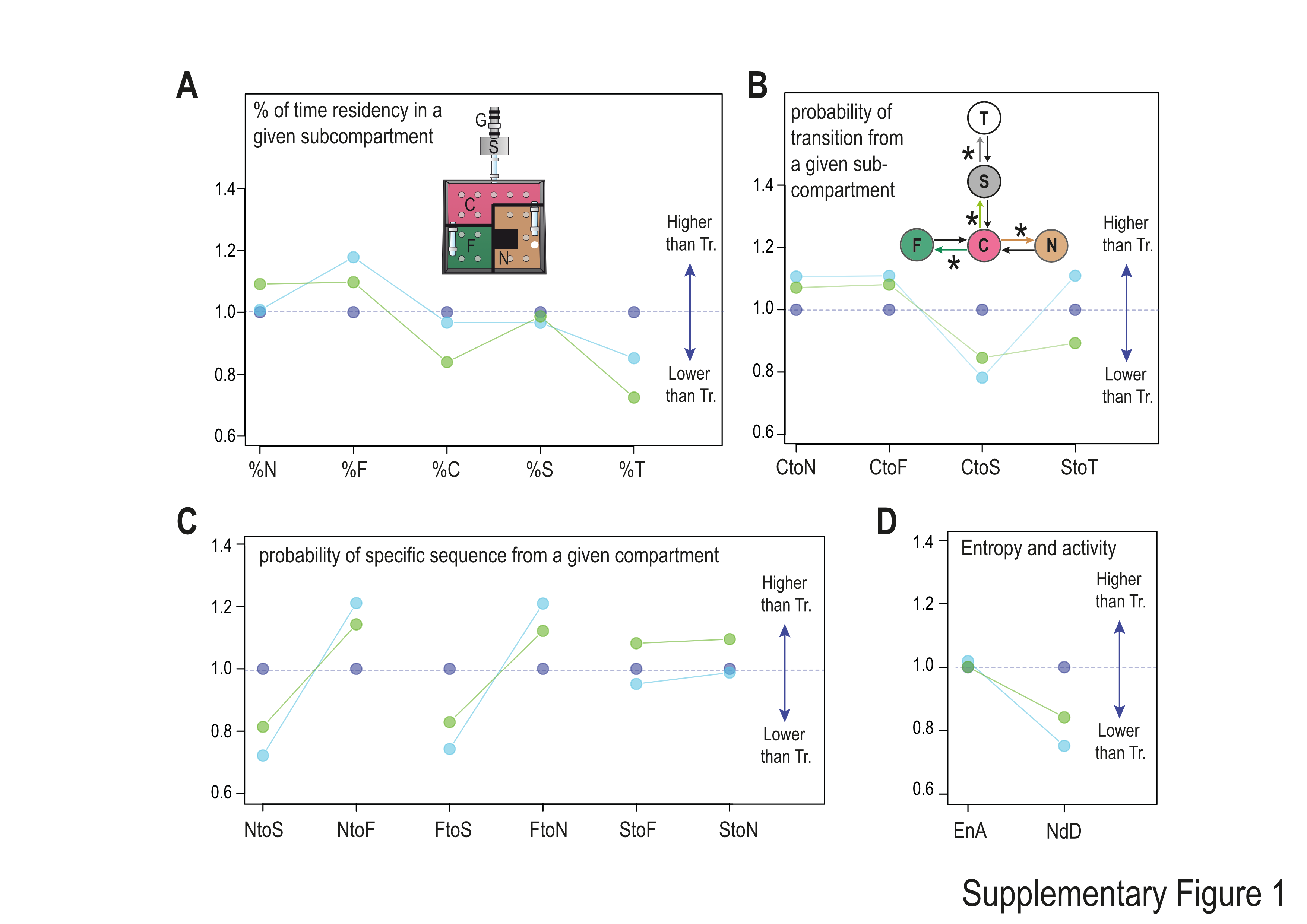

Supplement: S1 Fig — Data are normalized to mean Tracker value (Tr). (A) variation in Occupancy, i.e., from left to right percent time in Nest compartment (%N), percent time in Food compartment (%F), percent time in Center compartment (%C), percent time in Stair compartment (%S), percent time in T-Maze compartment (%T). (B) First order probability transitions. Inset: Transitions of interest are labeled with a star. Center to Nest (CtoN), Center to Food (CtoF), Center to Stair (CtoS), Stair to T-maze (StoT). (C) Second order transition (probability): Nest to Stair (NtoS), Nest to Food (NtoF), Food to Stair (FtoS), Food to Nest (FtoN), Stair to Food (StoF), Stair to Nest (StoN). (D) Activity levels: Entropy (EnA), Number of Detections (NbD). (TIFF) [file pbio.3002850.s001.tiff]

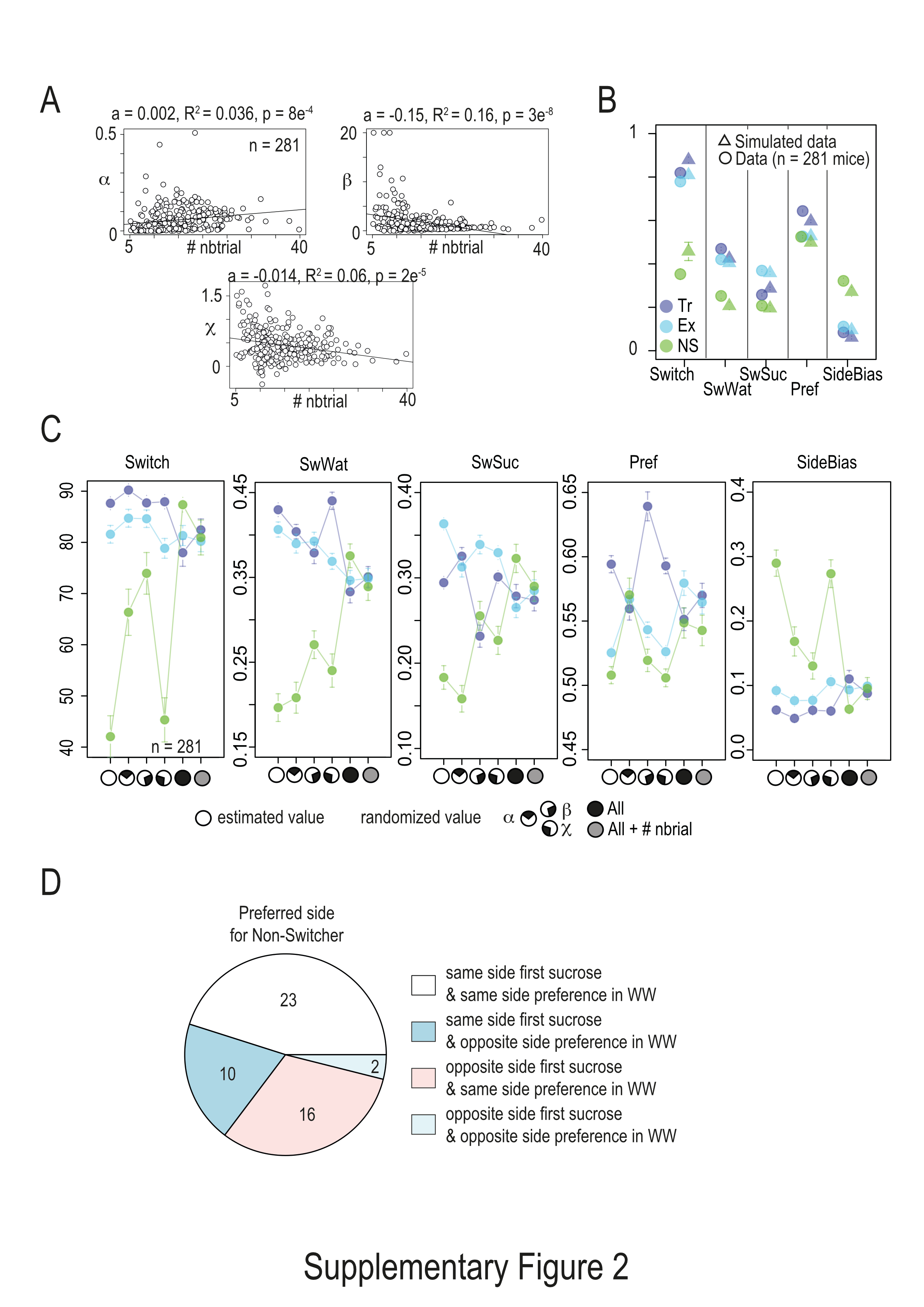

Supplement: S2 Fig — (A) Correlation (linear regression, a indicated the slope estimate, R2 the Adjusted R-squared and p the p-value) between the mean number of T-maze entry per day (# nbtrial) and χ (Top Left), β (Bottom), and α (Top right) (n = 281 mice). (B) Comparison of the mean of 5 variable (Switch, SwWat, SwSuc, Pref, SideBias) for Tr, Ex, and NS archetype obtained for data (o) and for a model sequence (Δ) of 300 choices (6 sessions of 50 choices) simulated with fitted values of α, β, and χ. (C) Attribution study in which each of the latent variables is manipulated independently from the other to assess its contribution to the Switch, SwWat, SwSuc, Pref, SideBias variables, for the 3 archetypes. The legend symbols represent different simulation conditions. We modeled 6 sessions of 50 choices for the simulations, except for the last condition where the number of choices was defined experimentally for each mouse. In this case, we defined the number of choices by taking the average number of trials per day for each mouse and multiplying it by 3 to represent 1 session of 3 days. Six such sessions were then used in the simulation. Empty circle: Each mouse is simulated using its estimated values of latent variables α, β and χ. Black circle: Each mouse is simulated with a random selection of latent variables α, β, and χ. Gray circle: Same as Black circle but with a number of trials/sessions estimated experimentally. White and Black circle: Each mouse is simulated with two of its estimated values and one is chosen randomly from the corresponding latent variables, respectively, α, β, or χ. (D) Percentage of Non-Switcher mice (n = 52) with a preferred side in WS session that correspond to: (white) same first sucrose choice side in WS session and same side preference in WW session, (blue) same first sucrose choice side in WS session and opposite side preference in WW session, (pink) opposite first sucrose choice side in WS session and same side preference in WW session and (light blue) opp [file pbio.3002850.s002.tiff]
